# Rectal gel application of *Withania somnifera *root extract expounds anti-inflammatory and muco-restorative activity in TNBS-induced Inflammatory Bowel Disease

**DOI:** 10.1186/1472-6882-11-34

**Published:** 2011-04-28

**Authors:** Pankaj Pawar, Suhit Gilda, Siddhesh Sharma, Suresh Jagtap, Anant Paradkar, Kakasaheb Mahadik, Prabhakar Ranjekar, Abhay Harsulkar

**Affiliations:** 1 Poona College of Pharmacy, Bharati Vidyapeeth University, Erandwane, Pune 411 023, India; 2 Interactive Research School for Health Affairs, Bharati Vidyapeeth University, Pune-Satara Road, Pune 411 043, India

## Abstract

**Background:**

Inflammatory Bowel Disease (IBD) is marked with chronic inflammation of intestinal epithelium driven by oxidative stress. Traditional treatments with plant extracts gained renewed interest due to their ability to ameliorate the multi factorial conditions like inflammation. We investigated the beneficial effects of *Withania somnifera *in Trinitro Benzyl Sulfonic Acid (TNBS) induced experimental IBD through a rectally applicable formulation.

**Methods:**

The study included (i) preparation of gel formulation from aqueous *Withania somnifera *root extract (WSRE), (ii) biochemical assays to determine its performance potential, (iii) testing of formulation efficacy in TNBS-induced IBD rat model, and (iv) histo-patholgical studies to assess its healing and muco-regenerative effect in IBD-induced rats. For this purpose, concentration dependant antioxidant activity of the extracts were evaluated using biochemical assays like (a) inhibition of lipid peroxidation, (b) NO scavenging, (c) H_2_O_2 _scavenging, and (d) ferric reducing power assay.

**Results:**

The extract, at 500 μg/ml, the highest concentration tested, showed 95.6% inhibition of lipid peroxidation, 14.8% NO scavenging, 81.79% H_2_O_2 _scavenging and a reducing capacity of 0.80. The results were comparable with standard antioxidants, ascorbic acid and curcumin. WSRE treatment positively scored on histopathological parameters like necrosis, edema, neutrophil infiltration. The post treatment intestinal features showed restoration at par with the healthy intestine. In view of these results, gel formulation containing an aqueous extract of *W. somnifera*, prepared for rectal application was tested for its anti-inflammatory activity in TNBS-induced rat models for IBD. Commercially available anti-inflammatory drug Mesalamine was used as the standard in this assay.

**Conclusions:**

Dose of the rectal gel applied at 1000 mg of WSRE per kg rat weight showed significant muco-restorative efficacy in the IBD-induced rats, validated by histo-pathological studies.

## Background

*Withania somnifera *(Dunal), also known as Ashwagandha, is an important member of family Solanaceae, utilized as a medicine for more than 2500 years in Indian medicinal classic "Ayurveda" [1]. Roots of this plant are considered most active for therapeutic purposes by virtue of significant accumulation of active constituents, withanolides [2]. Several withanolides with cyclooxygenase inhibitory and lipid peroxidation inhibitory activity were isolated from leaves and fruits of *W. somnifera *[3,4], besides anti-inflammatory [5], anti-tumor [6] and antioxidant [7] activities. Immunomodulatory role of *W. somnifera *roots and anti-inflammatory activity using adjuvant-induced arthritic rat models was also demonstrated [8,9]. Luvone et al [10] had observed that methanolic extracts of *W. somnifera *roots turns-on the synthesis of inducible nitric oxide synthase expression by acting at transcriptional level resulting in increased production of NO by macrophage, which was attributed to immunomodulatory and anti-inflammatory activity. Considering the various biological activities, roots of *W. somnifera *can potentially be utilized for the effective treatment of various inflammatory conditions.

Inflammatory bowel disease (IBD) encompasses (i) various conditions that result in chronic inflammation of small and/or large intestine, alike in young, adults and old, (ii) Ulcerative Colitis (UC), characterized by chronic mucosal and sub-mucosal inflammation of large intestine and rectum and (iii) Crohn's Disease (CD), which is chronic transmural inflammation of all/any part of the gastro-intestinal tract involving mucosa, sub-mucosa, muscular and connective tissue [11]. However, IBD, UC and CD are considered together because of similarities in their characteristics, pathology, complications, investigations, and treatment [12].

Etiology of IBD largely remains unknown [13]; however, development of tissue injury is attributed largely to distorted immune system and reactive oxygen species (ROS) [14]. Sustained production of reactive oxygen and nitrogen species is due to dysfunction of immune system is believed to play an important role in the development of intestinal colon injury [15]. It is therefore, logical to treat this disorder by controlling oxidative stress. Logically, antioxidant chemicals have been the main therapeutic strategy in IBD for last 50 years [16]. The popular treatment of IBD involves use of (a) 5-aminosalicylate based compounds, which are potent ROS scavengers [17], (b) broad spectrum antibiotics, (c) steroids and immuno-suppressants. These drugs are typically associated with side effects like nausea, anorexia, dizziness, headache, cytopenia, fever, myalgia, abnormal liver function, pleropericarditis, renal insufficiency and pulmonary toxicity. Plants are considered to be a goldmine to tackle complex inflammatory conditions with minimum of side effects. The effective herbs with antioxidant activity have been recently reviewed [18]. Numbers of reports are available on anti-inflammatory activities of plant-derived crude extracts or compounds derived from different plants. For example, curcumin has been widely investigated for the treatment of experimental colitis to decrease inflammation in therapeutic dosages [19-22]. Similarly, formulation containing a mixture of four herbal drugs (*Aegle marmeloes*, *Coriandrum sativam*, *Cyperus rotundus *and *Vetiveria zinzaniods*) showed significant inhibitory activity comparable with the standard drug prednisolone [12] against IBD induced in experimental animal models. It has become, therefore, imperative to search for safer alternative strategies that modulate the entire inflammatory pathway.

*Withania somnifera *is a unique plant where a wide range of activities has been demonstrated including antagonism with several pro-inflammatory factors and immunomdulation. In Ayurveda, enema of *W. somnifera *water extract is prescribed for intestinal ulcers, irritable bowel and rectal bleeding. All these attributes encouraged us to investigate its usefulness in the treatment of IBD [23]. Ours is the first report where aqueous extract of *W. somnifera *roots was assessed in Trinitro Benezyl Sulfonic Acid (TNBS) induced IBD in rat model, using formulation designed for rectal application. Mucosal adhesion of the formulation facilitated precise application of the extract on intestinal lesions.

## Methods

### Plant material and extract preparation

Authenticated roots of *W. somnifera *were procured from Green Pharmacy, Pune. Its aqueous extract was prepared by suspending 15 gm root powder in 100 ml distilled water, followed by shaking (150 rpm, at 60°C, for 24 h). The extract was filtered and clear suspension utilized for the antioxidant assays. Standardization of the extract was done by HPTLC method for quantification of Withanolide D using standard lab protocol.

### Antioxidant assays

Five different biochemical assays were undertaken to assess the antioxidant activity of *W. somnifera *aqueous roots extract (WSRE)

#### 1. Anti-lipid peroxidation activity

Anti-lipid peroxidation potential of WSRE was determined by estimating the inhibition of thiobarbituric acid reactive species (TBARS). Lipid peroxidation was initiated by adding 100 μl of 1 mM ferric chloride in 10% w/v colon tissue homogenate. Malonyldialdehyde (MDA) generated by the oxidation of polyunsaturated fatty acids upon reaction with two molecules of thiobarbituric acid (TBA) yielded a pink red complex, which was measured at 532 nm, from which % inhibition of lipid peroxidation by WSRE was calculated. In practice, mixtures containing 0.5 ml of tissue homogenate, 1 ml 0.15 M KCl and 0.5 ml different concentrations of WSRE were prepared. Lipid peroxidation was initiated by adding 100 μl of 1 mM ferric chloride. Incubated at 37°C for 30 min. The reaction was stoped by adding 2 ml ice-cold 0.25 N HCl (containing 15% trichloroacetic acid (TCA), 0.38% TBA and 0.2 ml 0.05% butylated hydroxyl toluene (BHT)). The reaction mixture was heated at 80°C for 60 min, cooled, centrifuged (5000 g ≈6900 rpm, 15 min). Absorbance of the supernatant was measured at 532 nm against a blank, which contained all reagents except colon homogenate and WSRE. Identical experiments were performed to determine the normal (without drug and FeCl_3_) and induced (with the drug) lipid peroxidation level in the tissue. The percentage of anti-lipid peroxidation effect (%ALP) was calculated by the following formula [24].

Where, A_FeCl3_: Absorbance of FeCl_3_, A_Normal_: Absobance of control reaction,

A_test_.: Absorbance of test reaction

#### 2. Nitric oxide (NO) scavenging activity

Nitric oxide radical inhibition was estimated using Griess-Illosvoy reaction principle. In this protocol, a reaction mixture (6 ml) containing sodium nitroprusside (10 mM, 2 ml), phosphate buffer saline (PBS) (0.5 ml) and different concentrations of extract or standard solution was prepared. A reaction control without test compound but equivalent amount of methanol was maintained. Incubation was carried out at 25°C for 150 min, followed by treating 0.5 ml of the reaction mixture with 1 ml Griess reagent A. In the next step, 1 ml of Griess reagent B was added and incubated at 25°C for 60 min. Absorbance was measured at 540 nm. The % inhibition was calculated using the formula [25].

Where A_cont _- absorbance of the control reaction

A_test _- absorbance of reaction with the extract.

#### 3. Hydrogen peroxide scavenging activity

Ability of the extract to scavenge H_2_O_2 _was determined as per Ruch et al [26]. A solution of H_2_O_2 _was prepared in PBS (pH 7.4) and its concentration determined spectrophotometrically. The reaction mixture (0.9 ml) containing extract (0.3 ml) and H_2_O_2 _(0.6 ml each) in PBS were incubated at ambient temperature for 10 min and its absorbance at 260 nm was determined 10 min later against a blank solution containing PBS without H_2_O_2_. The % H_2_O_2 _scavenging of both, the extract and standard were calculated as follows:

Where, A _cont_.: Absorbance of control reaction, A _test_: Absorbance of test reaction.

#### 4. Evaluation of the reducing power

The reducing power of the extract was measured by the transformation of Fe^3+ ^to Fe^2+ ^in the presence of different concentrations of extract at 700 nm as per Mau [27]. It involved mixing (a) 1 ml aliquot of different concentrations of WSRE (250-2500 μg/ml) with 2.5 ml phosphate buffer (pH 6.8) and 2.5 ml potassium ferricyanide, (b) incubating it at 50°C for 20 min, (c) arresting the reaction by addition of 2.5 ml TCA (d) centrifuging at 3000 rpm for 10 min, (e) taking 2.5 ml supernatant, diluting with 2.5 ml distilled water, (f) adding 0.5 ml of FeCl_3 _solution, and (g) measuring absorbance at 700 nm. Increased absorbance of the reaction mixture indicates increased reducing power.

### Induction of experimental colitis

The animal experimental procedures were in accordance with the regulation of institutional animal ethical committee, Poona College of Pharmacy, Bharati Vidyapeeth University, (CPCSEA/47/2007-08). Wistar rats weighing approximately 200-250 g each of either sex were distributed in four different groups (n = 6), i.e. healthy control, negative control, positive control and test group were housed in institutional animal house with controlled temperature (25°C). TNBS/ethanol induced colitis in the rat was established according to Elson et al [28]. After overnight fasting, each rat was lightly anaesthetised with diethyl ether, and a polyethylene cannula (4 mm diameter) was inserted into the lumen of the colon and advanced so that its tip was 6-8 cm proximal to the anus. Initially, each rat was lavaged with 2 ml of saline for enema followed by manual palpation of the abdomen to remove the fecal matter, if any. Next, TNBS (100 mg/kg of rat weight) dissolved in 50% ethanol (v/v) was instilled into the colon lumen (only 0.25 ml) through the rubber catheter, and the rat was maintained in a head-down position for 20 seconds to limit the expulsion of solution. From the fourth day up to 14^th ^day treatment was given by rectal administration of thermoreversable gel. Animals were maintained on water and diet *ad libitum *through out.

### Preparation of gel for rectal application

The gel preparation involved (a) use of sufficient amount of pluronic F127 to yield desired 20 (% w/w) gel, slowly added to cold (5°C) water with constant stirring, and (b) refrigeration of dispersion until a clear solution was formed. Concentration of WSRE was maintained such as to deliver 1 gm/kg body weight of the IBD-rats per day (effective concentration of withanolide D in the formulation was 0.0336% w/w) [29].

### Assessment of severity of colitis

At the end of the experiments, rats were sacrificed by cervical dislocation, the colon excised, opened longitudinally, and washed in saline. Macroscopic damage was assessed on the basis of semi-quantitative scoring system [30], which takes into account the area of inflammation and presence/absence of ulcers as described by Ukil et al [20] (No ulcer, no inflammation: 0; No ulcer, local hyperemia: 1; Ulceration without hyperemia: 2; Ulceration and inflammation at one site only: 3; Ulceration and inflammation at two or more sites: 4; and Ulceration extending more than 2 cm: 5). A 10 cm segment of colon was excised and weighed as an increase in weight is seen after induction of colitis. Further, these weights were compared with different groups to give an idea about weight recovery and malondialdehyde levels determined in each tissue, which served as an indicator of lipid peroxidation.

### Microscopic assessment of colitis

Histo-pathological studies of the colon were carried out at Local Pathology Laboratory, by an expert cyto-histopathologist by staining the sections with haematoxylin and eosin and taking their colored microscopic images, with resolution 10X-45X using a trinocular camera.

### Statistical analysis

Results are expressed as mean ± standard deviation of n observations. Analysis of variance (P < 0.05) to determine the statistical significance of inter-group comparisons, was considered statistically significant. Macroscopic and microscopic scores for colon erosion for the *W. somnifera *-pretreated groups were compared against those for the TNBS-treated group with a one-way ANOVA and Tukey test.

## Results

Antioxidant potential of aqueous extracts, determined biochemically using various assays, exhibited the following profiles.

### Anti-lipid peroxidation activity

Inhibitory effects of ascorbic acid and WSRE in the concentration range 100 to 500 μg/ml, on TBARS formed in rat colon induced by FeCl_3 _*in vitro *were studied. It was lowest (12.67%) at 100 μg/ml WSRE and gradually increased to 95.59% at 500 μg/ml, thus showing increase in the activity with an increase in the concentration. In ascorbic acid, anti-lipid peroxidation inhibition activity at 100 μg/ml was far higher (85.71%) than WSRE; it was six fold more than that of WSRE at the same concentration. It showed 99.97% inhibition of lipid peroxidation at 500 μg/ml (Table 1). Unpaired T-test showed significant P value of 0.0411.

**Table 1:** Comparative Antioxidant Free radical scavenging and anti-inflammatory activity of *Withania somnifera*

	% LPO Inhibition		% NO Scavenging	% H_2_O_2 _Scavenging	Reducing power	
**Conc. (μg/ml)**	***Withania somnifera***	**Ascorbic acid**	***Withania somnifera***	***Withania somnifera***	***Withania somnifera***	**Ascorbic acid**

100	12.67 ± 0.83	85.71 ± 0.72	10.55 ± 0.97	59.84 ± 0.79	0.4996 ± 1.34	0.4177 ± 1.53
200	51.73 ± 1.26	91.54 ± 0.98	14.30 ± 0.86	63.52 ± 0.85	0.5247 ± 1.21	0.5647 ± 1.04
300	65.02 ± 1.13	96.05 ± 0.86	14.41 ± 0.93	73.59 ± 0.98	0.6137 ± 0.95	0.6804 ± 1.02
400	75.70 ± 1.23	99.09 ± 0.84	14.75 ± 0.96	76.30 ± 1.13	0.7053 ± 0.89	0.7106 ± 1.21
500	95.59 ± 0.56	99.97 ± 1.36	14.77 ± 1.13	81.79 ± 1.23	0.7972 ± 0.58	0.8090 ± 0.96

### Nitric oxide scavenging activity

WSRE at 100 μg/ml concentration showed 10.55% NO scavenging activity, which increased to 14.30 at 200 μg/ml, beyond which no significant increase was noted with further increase in concentrations (IC_50 _= 58.70 μg/ml) (Table 1). Curcumin, used as a positive control at 50 μM concentration showed 11.79% NO scavenging, almost comparable with that by WSRE.

### Hydrogen peroxide scavenging activity

Ascorbic acid, used as a standard, at 10 μg/ml concentration showed 98.95% inhibition (data not shown in table). Hydrogen peroxide scavenging activity of the extract was concentration-dependent; about 59.84% H_2_O_2 _scavenging activity at 100 μg/ml concentration increased up to 81.79% as a function of increase in the concentration of the extract to 500 μg/ml (Table 1), indicating that aqueous extracts of roots of *W. somnifera *are good scavenger of peroxide radicals.

### Evaluation of the reducing potential

As expected from the literature, the WSRE exhibited significant reductive potential towards Fe^3+ ^to result into Fe^2+^. It was about 0.5 At 100 μg/ml concentrations, which was at par with the standard ascorbic acid. Further, it showed a clear dose dependent increase in reducing power (Table 1).

### Pharmacological screening

Rectal gel formulation was tested for its gelation and gel melting temperatures after addition of the extract. Bioadhesive strength of the formulation was measured on goat colon (Data not shown).

Malonyldialdehyde (MDA) levels were measured as an indicator of lipid peroxidation. In rats without any treatment (healthy control) average MDA levels were 11.63, far lower as compared to those in rats subjected to TNBS enema and no drug treatment (22.85), whereas rats treated with *W. somnifera *formulation (WSREF) (test control) showed significant decrease (18.15) in MDA levels, comparable with the mesalamine (standard drug) treated rats (18.66) (Table 2).

**Table 2:** Malondialdehyde levels as an indicator of lipid peroxidation

Lipid peroxidation in rat intestine	Healthy control	Positive control	Negative control	WSAE* treated
1	11.65	18.52	23.68	17.79

2	12.42	17.26	24.3	19.03

3	9.63	19.39	22.34	19.66

4	13.53	18.3	21.76	16.68

5	12.1	19.92	22.88	18.52

6	10.46	18.59	22.17	17.23

**AVG^#^**	**11.63**	**18.66**	**22.85**	**18.15**

STDV**	**1.401**	**0.920**	**0.969**	**1.125**

Abbreviation: * = *Withania somnifera *aqueous extract

# = Average

** = Standard deviation

### Macroscopic and histological evaluation

**Figure 1:**
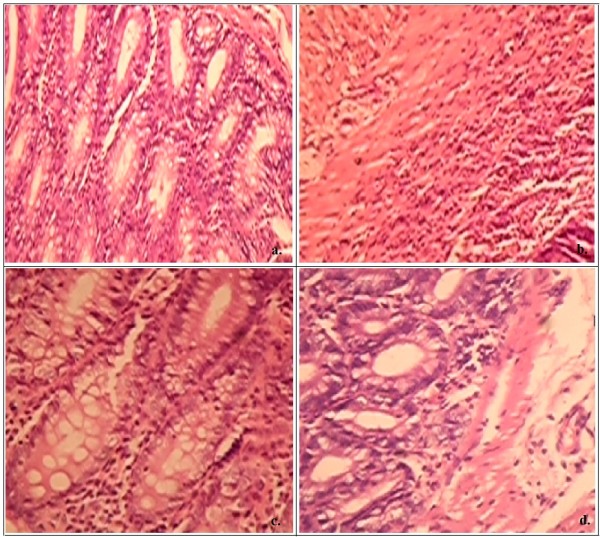
**Histopathological features of colon section of a). healthy control rat, b). rat with TNBS treatment, c). rat with TNBS +WSREF treatment, d). rat with TNBS+Mesalamine treatment**.

TNBS enema resulted in pronounced hemorrhagic and ulcerative lesions in the colon as observed after induction up to 8 days. Macroscopic examination of the affected colon and rectum revealed significant erosion of mucosal lining and severe ulceration often indicated as fecal occult blood. The histological features included (a) transmural necrosis, (b) edema and (c) diffused leucocytes infiltration of the sub-mucosa. Treatment of rats with WSREF (0.5 ml daily, containing 1 g/kg body weight) resulted in a significant decrease in the extent and severity of the injury of large intestine as evidenced by macroscopic damage score in Table 3 as well as histopathological assessment as in Figure 1. In healthy control (rats without any treatment), (i) intestinal crypts were evenly spaced, dipping down unto muscularis and (ii) *lamina propria mucosae *showed usual number of lymphocytes, plasma cells as well as eosinophils (Figure 1a). In negative control (rats treated with TNBS only), mucosa showed (i) ulceration, (ii) crypts were obligated, (iii) *lamina propria *marked with edema and (iv) infiltration by eosinophils as well as neutrophils (Figure 1b), while the test control (IBD induced rats treated with WSREF) mucosa showed (i) normal crypts, and (ii) *lamina propria *with usual number of mononuclear inflammatory cells (Figure 1c). In positive control (IBD-induced rats treated with mesalamine), mucosal crypts showed slight distortion and both edema and *lamina propria*, showed infiltration by neutrophils and eosinophils (Figure 1d).

**Table 3:** Effect of various treatments on macroscopic score analysis

Group name	Score rate						N*	Mean
			
	0	1	2	3	4	5		
HC**	5	1	0	0	0	0	6	0.16

NC	0	1	1	2	2	0	6	2.83

TC	0	3	2	1	0	0	6	1.60

PC	0	2	2	2	0	0	6	2.00

**HC: Healthy control; NC: colitis control; TC: Test control; PC: Positive control;

*n: number of rats per group.

**Figure 2:**
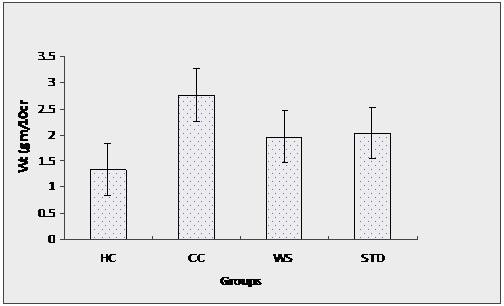
**Change in colon weight as a function of period of treatment**. HC: Healthy control, CC: Colitis control, WS: WSREF treated, STD: Standard (Mesalamine) control.

**Figure 3:**
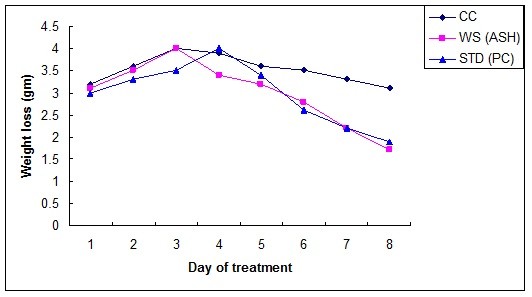
**Change in rat weight as a function of period of treatment**.

The observed inflammatory changes of the large intestine were associated with an increase in the weight of colon (Figure 2) as well as significant decrease in the body weight as compared to control rat (Figure 3). In contrast, no significant increase in the colon weight was found in the TNBS-treated rats on treatment with WSRE and marketed formulation of mesalamine. Moreover, treatment with these formulation dosages significantly reduced the loss in body weight, which correlated well with the amelioration of colon injury. The above data was subjected to statistical treatment by Tukey test and p values were obtained. The p value < 0.001 for *W. somnifera *and standard group was highly significant; suggesting that rats treated with *W. somnifera *rectal gel formulation showed marked recovery.

## Discussion

IBD is a common gastro-intestinal disorder marked with chronic inflammation of intestinal epithelium, damaging mucosal tissue and manifests into several intestinal and extra-intestinal symptoms, mainly related to oxidative stress, inflammation and autoimmune type.

TNBS-induced model is an experimental model of Th 1, like gut inflammation, mimics human Crohn's Disease and is widely used to investigate IBD [28]. TNBS enema develops hapten-induced delayed type hypersensitivity and results in the development of chronic colitis involving granuloma with infiltration of inflammatory cells in all layers of the intestine. This is due to TNBS-induced over expression of IL-12, IFN-γ and IL-2, which supports the study that inducible colitis is due to Th-type 1 response [31]. Ilan et al. [32] studied the involvement of immune system in the pathogenesis of IBD after observing the similarities between human disease and TNBS-induced colitis. Their study revealed that IBD could be considered as an imbalance between pro-inflammatory and anti-inflammatory mediators. Monocytes/macrophages, polymorphonuclear leucocytes (PMNs) and endothelial cells are mainly involved in inflammatory response and their activation forces them to aggregate and infiltrate the tissue, where they undergo respiratory burst, which increases their oxygen use resulting into oxidative damage to the tissue and triggers the production of pro-inflammatory cytokines, ROS and other mediators of inflammation [33].

Initiation and perpetuation of inflammatory cascade by ROS causes subsequent tissue damage through the activation of nuclear factor kappa B (NF-kB), which is a ubiquitous transcription factor involved in the regulation of several genes in immune and inflammatory responses [34]. Oxidants are potent activators of NF-kB while the groups of structurally diverse anti-oxidants of herbal origin are capable of inhibiting NF-kB activation [35]. Human body has its own anti-oxidant defense system, which involves enzymes such as superoxide dismutases, catalases and glutathione peroxidases. However, this enzymatic anti-oxidant defense system is often not sufficient, leading to increased free radicals and oxidative damage ultimately resulting in severe inflammation and cell death [36].

The gastro-intestinal tract is a major site of generation of pro-oxidants, whose production is primarily due to the presence of plethora of microbes, food ingredients and interaction between immune cells. Not surprisingly, ROS has been implicated in the initiation and perpetuation of inflammatory disorders and pathogenesis of IBD. In one study, saliva of IBD patients were studied as an indicator of existence of oxidative and nitrosative stress. Excessive NO as well as higher levels of epidermal growth factor were found associated with the disease [37]. Similarly increased ROS in colon mucosa of UC patients was demonstrated [38,39] and animal model exhibits increased oxidation and lipid peroxidation during initiation of colitis [40].

*W. somnifera *extract seems to be a balanced combination of biologically active ingredients as demonstrated by several workers. The cyto-protective and anti-inflammatory activity has protected experimental animals against the induced diseases. Antibacterial activity demonstrated by *W. somnifera *may also have significant role to play in IBD, especially for combating against the intestinal opportunistic pathogens known to play an important role in pathogenesis of IBD. Carminative and anti-diarrheal activity of *W. somnifera *may also be useful in restoring the disturbed gastro-intestinal mobility. Anti-oxidant activity is thought to play a central role in preventing inflammation as well restoration of mucosal lining. In the present study, aqueous extracts of *W. somnifera *roots have shown an excellent anti-oxidant activity, which is in total agreement with study carried out by Bhatnagar et al [41]. *W. somnifera *exhibits inhibition of (a) cyclooxygenase (b) activation of NF-kB induced inflammatory markers (like tumor necrosis factor alpha (TNF-αalpha) and interleukins) [42]. The withanolides constituting active ingredients of *W. somnifera *roots shows promising antibacterial, anti tumor, immunomodulating and anti-inflammatory properties [9].

Anti-ulcer activity of methanolic extract of *W. somnifera *and its action against stress pyloric ligation induced gastric ulcer in rats has been reported [41]. Treatment with *W. somnifera *extract (100 mg/kg/day p.o.) for 15 days, significantly reduced volume of gastric secretion, free total acidity and ulcer index as compared to control group. Significant increase in total carbohydrates (TC) and its ratio to total protein (TP) was also observed, without significant change in total proteins. A significant increase in antioxidant enzymes (viz. catalase, superoxide dismutase (SOD)) and decrease in malondialdihyde (MDA) was observed. Increased MDA levels after TNBS enema decreased upon treatment with *W. somnifera *formulation (Table 2). *W. somnifera *extract was found effective antiulcerogenic agent, comparable of ranitidine hydrochloride [41]. Treatment with *W. somnifera *formulation has shown decrease in macroscopic scores for the IBD. Histopathology examination of *W. somnifera *extract treated group revealed less damage compared to healthy animals (control group). Mesalamine treatment compared to *W. somnifera *formulation has shown significant and comparable protection in the rats in our study as revealed by the decreased colon weight and better gain in body weight. In the light of above properties, WSREF can potentially cure local inflammation, modulate immune system and can be a logical choice in relieving IBD symptoms. Administration of Iranian folk herbal medicines *Ziziphora clinopoides *and *Teucrium persicum *were reported to boost body's antioxidant mechanism such as SOD and catalase, with concomitant decrease in pro-inflammatory factors like TNF-αalpha, IL-1β. These studies suggest potential dexterity of folk medicine in management of disease like IBD where free radicals and inflammation is the major pathophysiology [43,44].

Success of herbal extracts in repairing tissue damage in experimental colitis could be enhanced by changing the route of administration. Several plant extracts have been reported beneficial in IBD, however, to our knowledge there is no report on a rectally applicable formulation. The pluronic rectal gel formulation impregnated with WSRE is in liquid form that is easy to apply as enema. At body temperature, it turns into a gel, which covers the rectum surface and due to its mucoadhesive property it forms a layer that persist and slowly release WSRE at the lesions in the rectum. This feature enhanced the beneficial effects of WSRE and resulted in reduced inflammation, faster healing and mucorestoration as revealed by the microscopic observations. Not all the extracts with good antioxidant activity could be beneficial in IBD. Harputluoglu et al [16] investigated oral application of *Gingko biloba *extract in acetic acid-induced colitis. Despite of an excellent antioxidant activity and antagonistic activity against platelets activating factor, (known to play a key role in pathogenesis of IBD), the extract failed to show noticeable recovery in experimental colitis. Based on the present data, topical application of aqueous extract of *W. somnifera *roots is strongly recommended in the treatment of distal colitis, especially for an early recovery in the damaged tissue. This data also substantiate the traditional way of treating colitis patients with enema of *Ashwagandha *extract. Detailed molecular study to identify the precise nature of active molecules will further assist in understanding their mechanism of action.

## Conclusions

This study affirms antioxidant potential of aqueous extract of roots of *Withania somnifera *and it's utility to ameliorate inflammation, which is the key pathology in IBD. The topical application in the form of rectal gel formulation proved to be as effective as the mesalamine treatment. In addition, WSREF showed marked muco-restoration and provides alternative strategy of practical significance for the treatment of Inflammatory Bowel Disease.

## Competing interests

The authors declare that they have no competing interests.

## Authors' contributions

PP, SG, SS and SJ carried out the study, designed experimental work, data collection and analysis. PP, SG and AH supervised the work and prepared the draft of manuscript. AP, KM and PK are involved evaluation of the data and corrected the manuscript. All the authors read and approved the final manuscript.

## Pre-publication history

The pre-publication history for this paper can be accessed here:

http://www.biomedcentral.com/1472-6882/11/34/prepub

## References

[B1] Bhattacharya A Ghosal S Bhattacharya SK Anti-oxidant effect of *Withania somnifera *glycowithanolides in chronic footshock stress-induced perturbations of oxidative free radical scavenging enzymes and lipid peroxidation in rat frontal cortex and striatum J Ethnopharmacol 2001 74 1 6 10.1016/S0378-8741(00)00309-3 11137343

[B2] Tripathy AK Shukla YN Kumar S *W. somnifera *[*Withania somnifera*] Dunal [Solanaceae]: A status report J Med Arom Plant Sci 1996 18 46 62

[B3] Jayaprakasam B Nair MG Cyclooxygenase-2 inhibitory withanolides from leaves of *Withania somnifera* Tetrahedron 2003 59 841 849 10.1016/S0040-4020(02)01601-0

[B4] Jayaprakasam B Strasburg GA Nair MG Potent lipid peroxidation inhibitors from *Withania somnifera *fruits Tetrahedron 2004 60 3109 3121 10.1016/j.tet.2004.01.016

[B5] Al-Hindwani MK Al-Khafaji SH Abdul-Nabi MH Anti-granuloma activity of Iraqi *Withania somnifera* J Ethnopharmacol 1992 37 113 116 10.1016/0378-8741(92)90069-4 1434685

[B6] Devi PU *Withania somnifera *Dunal [*W. somnifera*]: Potential plant source of a promising drug for cancer chemotherapy and radiosensitization Ind J Expt Biol 1996 34 927 932 9055640

[B7] Russo A Izzo AA Cardile V Borrelli F Vanella A Indian medicinal plants as antiradicals and DNA cleavage protectors Phytomed 2001 8 125 132 10.1078/0944-7113-00021 11315755

[B8] Rasool M Marylatha L Varalakshmi P Effect of *Withania somnifera *on lysosomal acid hydrolases in adjuvant-induced arthritis in rats Pharma Pharmacol Comm 2000 6 187 190 10.1211/146080800128735863

[B9] Rasool M Varalakshmi P Immunomodulatory role of *Withania somnifera *root powder on experimentally induced inflammation: An *in vivo *and *in vitro *study Vascular Pharmacol 2006 44 406 410 10.1016/j.vph.2006.01.015 16713367

[B10] Luvone T Esposito G Capasso F Izzo A Induction of nitric oxide synthase expression by *Withania somnifera *in macrophages Life Sci 2003 72 1617 1625 10.1016/S0024-3205(02)02472-4 12551750

[B11] Fiocchi C Inflammatory bowel disease: etiology and pathogenesis Gastroenterol 1998 115 183 205 10.1016/s0016-5085(98)70381-6 9649475

[B12] Jagtap AG Shirke SS Phadke AS Effect of polyherbal formulation on experimental models of inflammatory bowel diseases J Ethnopharmacol 2004 90 195 204 10.1016/j.jep.2003.09.042 15013181

[B13] Gurudu S Fiocchi C Katz JA Inflammatory bowel disease Best Pract Res Clin Gastroenterol 2002 16 77 90 10.1053/bega.2001.0267 11977930

[B14] Rezaie A Parker RD Abdollahi M Oxidative stress and pathogenesis of inflammatory bowel disease: an epiphenomenon or the cause? Dig Dis Sci 2007 52 2015 2021 10.1007/s10620-006-9622-2 17404859

[B15] Pavlick KP Laroux FS Fuseler J Wolf RE Gray L Hoffman J Grisham MB Role of reactive metabolites of oxygen and nitrogen in inflammatory bowel disease Free Rad Biol Med 2002 33 311 322 12126753 10.1016/s0891-5849(02)00853-5

[B16] Harputluoglu M Demirel U Yucel N Karadag N Temel I Firat N Ara C Aladağ M Karincaoğlu M Hilmioğlu F The effect of *Gingko biloba *extract on acetic acid on acetic acid induced colitis in rats Turk J Gastroenterol 2006 17 177 182 16941250

[B17] Miles AM Grisham MB Antioxidant properties of amino-salicylates Methods Enzymol 1994 234 555 572 7808332 10.1016/0076-6879(94)34128-1

[B18] Rahimi R Mozaffari S Abdollahi M On the use of herbal medicines in management of inflammatory bowel diseases: a systematic review of animal and human studies Dig Dis Sci 2009 54 471 480 10.1007/s10620-008-0368-x 18618255

[B19] Sugimoto K Hanai H Tozawa K Aoshi T Uchijima M Nagata T Koide Y Curcumin prevents and ameliorates trinitrobenzenersulfonic acid-induced colitis in mice Gastroenterol 2002 123 1912 1922 10.1053/gast.2002.37050 12454848

[B20] Ukil A Maity S Karmarkar S Datta N Vedasiromoni JR Das PK Curcumin, the major component of food flavour turmeric, reduces mucosal injury in trinitrobenzene sulphonic acid-induced colitis Br J Pharmacol 2003 139 209 218 10.1038/sj.bjp.0705241 12770926 PMC1573841

[B21] Salh B Assi K Templeman V Parhar K Owen D Gomez-Munoz A Jacobson K Curcumin attenuates DNB-induced murine colitis Am J Physiol Gastrointest Liver Physiol 2003 285 235 243 10.1152/ajpgi.00449.2002 12637253

[B22] Jian YT Mai GF Wang JD Zhang YL Luo RC Fang YX Preventive and therapeutic effects of NF-kappa B inhibitor curcumin in rats colitis induced by trinitrobenzene sulfonic acid World J Gastroenterol 2005 1 1747 1752 10.3748/wjg.v11.i12.1747 PMC4305867 15793857

[B23] Shastry MK Yadava RK Singh RH Effect of vasti therapy in the management of irritable bowel syndrome (Pakwasayagata vata Vyadhi) J Res Ayurveda Siddha 1996 17 16 19

[B24] Wade J Van R Quantitation of malonaldehyde [MDA] in plasma, by ion-pairing reverse phase high performance liquid chromatography Biochem Med 1985 33 291 296 10.1016/0006-2944(85)90003-1 4015630

[B25] Sreejayan N Rao MNA Nitric oxide scavenging by curcuminoids J Pharm Pharmacol 1997 49 105 107 9120760 10.1111/j.2042-7158.1997.tb06761.x

[B26] Ruch RJ Cheng SJ Klaunig JE Prevention of Cytotoxicity and inhibition of intracellular communication by antioxidant catechins isolated from Chinese green tea Carcinogenesis 1989 10 1003 1008 10.1093/carcin/10.6.1003 2470525

[B27] Mau EL Antioxidant properties of several medicinal mushrooms J Agri Food Chem 2002 50 6072 6077 10.1021/jf0201273 12358482

[B28] Elson CO Sartor RB Tennyson GS Riddell RH Experimental models of inflammatory bowel disease Gastroenterol 1995 109 1344 1367 10.1016/0016-5085(95)90599-5 7557106

[B29] Wallace JL Kinnan CM An orally active inhibitor of leukotriene synthesis accelerates healing in a rat model of colitis Am J Physiol 1990 258 527 534 10.1152/ajpgi.1990.258.4.G527 1970708

[B30] Elson CO Beagley KW Sharmanov AT Fujihashi K Kiyono H Tennyson GS Cong Y Black CA Ridwan BW McGhee JR Hapten-induced model of murine inflammatory bowel disease: mucosal immune responses and protection by tolerance J Immunol 1996 157 2174 2185 8757344

[B31] Neurath M Fuss I Kelshall B Stuber E Strober W Antibodies to interleukin 12 abrogate established experimental colitis in mice J Expt Med 1995 182 1281 1290 10.1084/jem.182.5.1281 PMC2192205 7595199

[B32] Ilan Y Weksler-Zangen S Ben-Horin S Diment J Sauter B Rahbani E Engelhardt D Chowdhury NR Chowdhury JR Goldin E Treatment of experimental colitis by oral tolerance induction: A central role for suppressor lymphocytes Am J Gastroenterol 2000 95 966 973 10.1111/j.1572-0241.2000.01935.x 10763946

[B33] Devise MG Hagen PO Systemic inflammatory response syndrome Br J Surg 1997 84 920 935 10.1002/bjs.1800840707 9240130

[B34] Siebenlist U Franzoso G Brown K Regulation and function of NF-kappa B Annual Rev Cell Biol 1994 10 405 455 10.1146/annurev.cb.10.110194.002201 7888182

[B35] Schreck R Albermann K Baeuerle PA Nuclear factor kappa B: an oxidative stress-responsive transcription factor of eukaryotic cells [a review] Free Radic Res Commun 1992 17 221 237 10.3109/10715769209079515 1473734

[B36] Halliwell B Free radicals, anti-oxidants and human diseases: curiosity, cause or consequence Lancet 1994 344 721 724 10.1016/S0140-6736(94)92211-X 7915779

[B37] Jahanshahi G Motavasel V Rezaie A Hashtroudi AA Daryani NE Abdollahi M Alterations in antioxidant power and levels of epidermal growth factor and nitric oxide in saliva of patients with inflammatory bowel diseases Dig Dis Sci 2004 49 1752 1757 10.1007/s10620-004-9564-5 15628697

[B38] Keshavarzian A Sedghi S Kanofsky J List T Robinson C Ibrahim C Winship D Excessive production of reactive oxygen metabolites by inflammed colon: Analysis by chemi-luminescence probe Gastroenterol 1992 103 177 185 10.1016/0016-5085(92)91111-g 1612325

[B39] Simmonds NJ Allen RE Stevens TR van Someren RN Blake DR Rampton DS Chemiluminescence assay of mucosal reactive oxygen metabolites in inflammatory bowel disease Gastroenterol 1992 103 186 196 10.1016/0016-5085(92)91112-h 1319369

[B40] Grisham MB Volkmer C Tso P Yamada T Metabolism of trinitrobenzene sulfonic acid by the rat colon produces reactive oxygen species Gastroenterol 1991 101 540 547 10.1016/0016-5085(91)90036-k 1648528

[B41] Bhatnagar M Sisodia SS Bhatnagar R Anti-ulcer and antioxidant activity of *Asparagus racemosa *WILD and *Withania somnifera *DUNAL in rats Ann N Y Acad Sci 2005 105 261 278 10.1196/annals.1352.027 16387694

[B42] Mishra LC Singh BB Dagenais S Scientific basis for the therapeutic use of *Withania somnifera *[Ashwagandha]: A review Alternative Med Rev 2000 5 334 346 10956379

[B43] Amini-Shirazi N Hoseini A Ranjbar A Mohammadirad A Khoshakhlagh P Yasa N Abdollahi M Inhibition of tumor necrosis factor and nitrosative/oxidative stresses by *Ziziphora clinopoides *(Kahlioti); a molecular mechanism of protection against dextran sodium sulfate-induced colitis in mice Toxicol Mech Methods 2009 19 183 189 10.1080/15376510701533996 19778264

[B44] Abdolghaffari AH Baghaei A Moayer F Esmaily H Baeeri M Monsef-Esfahani HR Hajiaghaee R Abdollahi M On the benefit of Teucrium in murine colitis through improvement of toxic inflammatory mediators Hum Exp Toxicol 2010 29 287 295 10.1177/0960327110361754 20144954

